# WICID 2.0: Integrating evidence and ethics in decisionmaking and guideline development on SARS-CoV-2

**DOI:** 10.1093/eurpub/ckac129.543

**Published:** 2022-10-25

**Authors:** L Arnold, S Bimczok, J Lehner, J Stratil

**Affiliations:** 1 Berlin, Germany; 2 Academy of Public Health Services, Angewandte ÖGD-Forschung und Transfer, Duesseldorf, Germany

## Abstract

Evidence-informed decision-making (EIDM) requires the balancing of numerous and often conflicting factors. During the SARS-CoV-2 pandemic, sound and fair EIDM procedures were challenged by time constraints and limited evidence. Beneficial effects had to be weight against public health impacts beyond COVID-19, broad societal consequences, or individual liberties. Evidence-to-decision (EtD) frameworks are neither able nor intended to replace stakeholder participation, but can serve as a tool to ensure the relevance and completeness of criteria to be considered for EIDM in public health and guideline development. Employing ‘best-fit’ framework synthesis, we used the WHO-INTEGRATE framework as a starting point to develop the WHO-INTEGRATE COVID-19 framework version 1.0. WICID 1.0 is based on a content analysis of comprehensive strategy documents to guide policy makers in implementing new or decrease existing measures to protect against COVID-19 in Germany. WICID 1.0 was validated by coding the framework against an updated set of the key strategy documents, and key strategy documents addressing non-pharmacological measures in long-term care facilities. In total, 12 key strategy documents were analysed to develop WICID 1.0, and 18 + 23 documents were analyzed for its refinement towards WICID 2.0. The revised framework consists of 11 + 1 criteria and includes implications for the health of individuals and populations due to and beyond COVID-19, infringement on liberties and fundamental human rights, acceptability and equity considerations, societal, environmental and economic implications, as well as implementation, resource and feasibility considerations. Validation found high consistency with minor revisions between WICID 1.0 and 2.0. WICID can be a tool to support researchers, practitioners, and policy makers to systematically integrate evidence and ethics and to balance of health, societal and other considerations when reflecting on PH interventions targeting COVID-19.

**Key messages:**

• Due to the rapidly developing pandemic, decision-making process often did not include the views of all affected stakeholders and did not adequately include all criteria and considerations of relevance.

• The WICID Framework can serve as a tool to support decision-makers in accounting for relevant considerations and criteria, even when not all stakeholders could be included.

